# Urgent Rectus Sheath Hematoma Induced by Severe Coughing

**DOI:** 10.31662/jmaj.2025-0229

**Published:** 2026-02-13

**Authors:** Kazuhiko Iwasaki, Satoshi Watanabe, Seiji Yano

**Affiliations:** 1 Department of Internal Medicine, Anamizu General Hospital, Ho-su gun, Ishikawa, Japan; 2 Department of Respiratory Medicine, Kanazawa University Graduate School of Medical Sciences, Kanazawa, Ishikawa, Japan; 3 Department of Respiratory Medicine, Kaga Medical Center, Kaga, Ishikawa, Japan

**Keywords:** rectus sheath hematoma, severe coughing, skin discoloration

## Abstract

Rectus sheath hematoma (RSH) is an uncommon but clinically significant cause of acute abdominal pain, most often associated with anticoagulant therapy, abdominal trauma, or prior surgery. Although coughing is a recognized but rare etiology, cases without anticoagulant exposure are unusual. We report the case of a 57-year-old man with hypertension who presented with acute right abdominal pain after 1 week of persistent cough. Physical examination revealed abdominal wall discoloration, a firm mass, and a positive Carnett sign, suggesting an abdominal wall origin of pain. Laboratory evaluation showed leukocytosis and elevated inflammatory markers, and computed tomography demonstrated bronchopneumonia and multiple hematomas within the right rectus abdominis muscle. The hematomas showed high attenuation, measuring 50-75 Hounsfield units, consistent with acute hematoma. Despite initial hemodynamic stability, the patient rapidly deteriorated with tachycardia and hypotension, necessitating emergency hematoma evacuation. After intensive care and rehabilitation, he was discharged in good condition. This case highlights that cough-induced RSH, although rare, can progress rapidly to hemodynamic instability. Physicians should consider RSH in the differential diagnosis of abdominal pain with abdominal wall discoloration following coughing episodes, even in the absence of traditional risk factors, as timely recognition and intervention are essential to prevent morbidity and mortality.

## Introduction

Rectus sheath hematoma (RSH) results from bleeding into the rectus sheath, usually due to injury of the superior or inferior epigastric arteries or direct muscle tear. Although uncommon, it is an important diagnostic consideration in patients with acute abdominal pain, with an incidence of 0.6% in the general population and 6.6% in elderly patients receiving anticoagulation therapy ^(1), (2)^. RSH is commonly associated with anticoagulant or antiplatelet therapy, abdominal trauma, or prior surgery. However, it may also occur secondary to severe coughing, and cases in otherwise healthy adults without risk factors are rare ^(3), (4)^. We report a case of cough-induced RSH in a previously healthy man with bronchitis.

## Case Report

**Figure 1.**
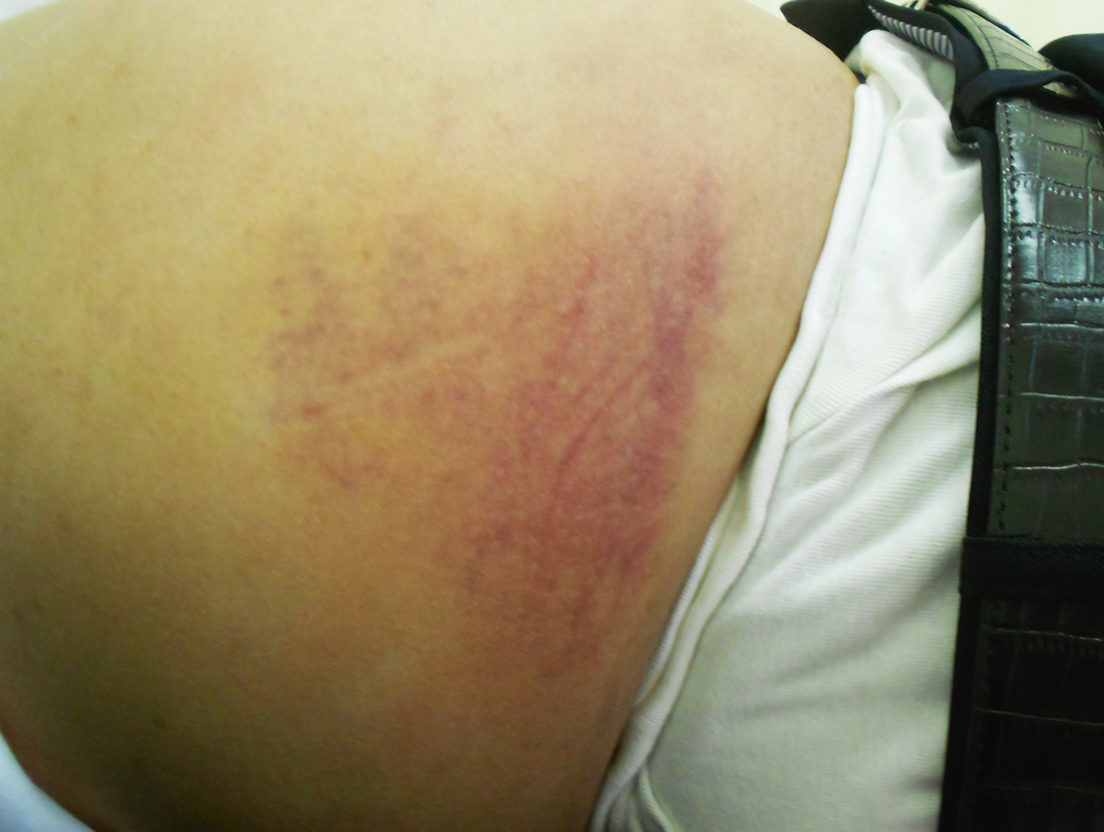
Skin discoloration with pain and swelling spanning from the lower abdomen to the right flank.

**Figure 2.**
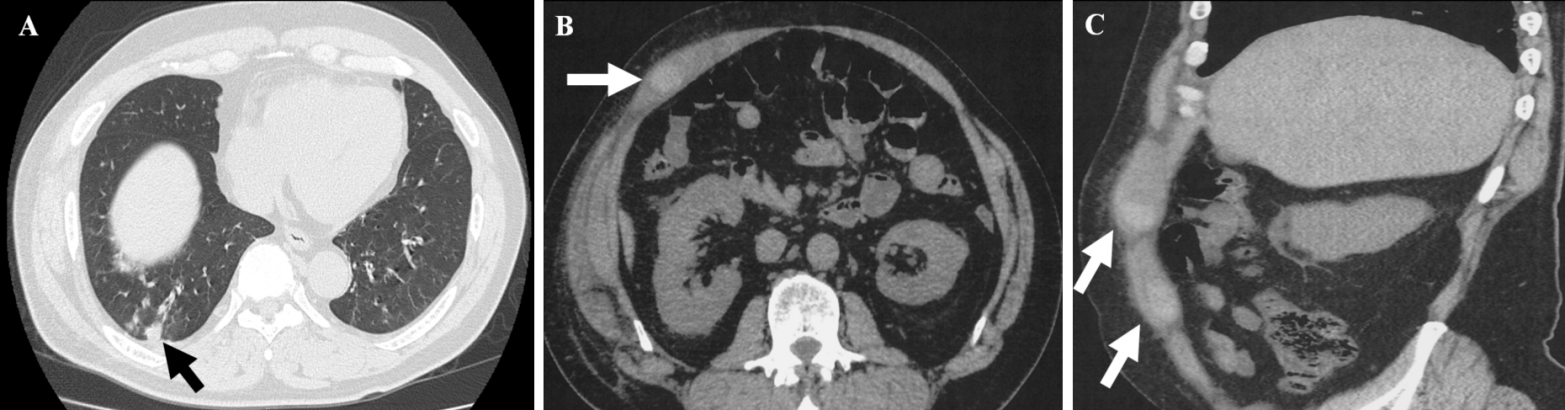
(A) Axial lung computed tomography (CT) scan showing right bronchopneumonia (arrow). (B) Axial abdominal CT scan showing hematomas (arrow). (C) Sagittal abdominal CT scan showing multiple hematomas (arrow).

A 57-year-old man with hypertension treated with amlodipine presented with acute right abdominal pain. He was a former smoker and had no history of surgery, anticoagulant, or antiplatelet therapy. He had experienced persistent cough for one week, and 2 days before presentation he developed right abdominal discomfort that acutely worsened. On arrival, his temperature was 37.3°C, blood pressure 146/97 mmHg, pulse 86/min, respiratory rate 16/min, and oxygen saturation 97% on room air. Physical examination revealed discoloration extending from the lower abdomen to the right flank and a firm, poorly defined, elastic mass on palpation. Carnett’s sign was positive, supporting the abdominal wall origin of pain (Figure 1). Laboratory results showed leukocytosis (white blood cell 15,200/μL) and elevated C-reactive protein (17.35 mg/dL), while renal and liver function and coagulation parameters were within normal limits, with no evidence of bleeding disorders, liver failure, or connective tissue disease. Computed tomography (CT) revealed bronchopneumonia and multiple hematomas within the right rectus abdominis muscle. The two dominant intramuscular hematomas measured 30 × 21 × 29 mm and 21 × 38 × 19 mm, corresponding to estimated volumes of approximately 18 mL and 15 mL, respectively. The hematomas demonstrated high attenuation, measuring 50-75 Hounsfield units, which is consistent with the density of acute hematoma (Figure 2A-C). During observation, the patient’s abdominal pain worsened, and he developed tachycardia with unstable hemodynamics; he was therefore transferred to a tertiary care hospital. After transfer, contrast-enhanced CT showed possible enlargement of the intramuscular hematomas with extension throughout the right rectus abdominis muscle, and the overlying skin discoloration had progressed. These dynamic radiological and clinical changes contributed to the decision to proceed with urgent surgical evacuation. The anterior rectus sheath was incised, and multiple intramuscular hematomas containing dark-red clots were evacuated after intraoperative samples were obtained for culture; the cavity was irrigated with saline, and local hemostasis was achieved. Because of the presence of multiple hematomas, a single responsible artery could not be clearly identified. Finally, the fascia and subcutaneous tissue were closed and a drain was placed. Postoperatively, the patient was admitted to the intensive care unit for close monitoring and respiratory support. He required high-flow nasal cannula oxygen therapy but not invasive mechanical ventilation and remained in the intensive care unit for 2 days before transfer to the general ward. The wound was managed with negative-pressure therapy and later skin grafting, and he was discharged after about 1 month. Sputum cultures grew *Haemophilus influenzae*, and the lower lobe pneumonia was considered the source of fever. In contrast, intraoperative cultures from the hematoma were negative, and there were no CT findings suggestive of an infected hematoma, indicating that the rectus sheath hematomas were not infected.

## Discussion

RSH is an uncommon but important condition that can mimic other causes of acute abdomen. The mean age of onset ranges from 45 to 70 years and it is more common in women, likely due to reduced rectus muscle mass ^(5)^. It occurs when bleeding develops within the rectus sheath, most often due to rupture of the superior or inferior epigastric arteries ^(6)^.

Cough-induced RSH has been documented and represents a minority of cases. Mechanical stress such as coughing, vomiting, or abrupt movement can precipitate rupture of the epigastric vessels ^(7)^. Our patient had persistent cough and no anticoagulant exposure, highlighting this rare etiology.

RSH usually presents with acute abdominal pain and a palpable abdominal wall mass, sometimes accompanied by abdominal wall discoloration (Cullen’s or Grey Turner’s sign). The Carnett sign, in which abdominal tenderness remains the same or worsens when the patient tenses the abdominal muscles, and the Fothergill sign, in which a palpable mass remains fixed with contraction of the rectus muscle, help localize pain to the abdominal wall rather than intra-abdominal organs ^(8)^. Other reported symptoms include nausea (23%), vomiting (15%), and fever or chills ^(7)^.

For diagnosis, CT is the gold standard, allowing accurate identification of hematoma size, location, and extension ^(6)^. CT angiography can additionally reveal active extravasation, an important predictor of the need for intervention. Most RSHs can be managed conservatively with analgesia, rest, monitoring, and reversal of coagulopathy, but several predictors of conservative management failure have been reported, including large hematoma volume, rapid hemoglobin decline, high transfusion requirement, and active extravasation on CT angiography ^(9), (10)^. Endovascular embolization is increasingly used as a minimally invasive option ^(6)^. In our patient, hemodynamic instability together with radiological concern for enlargement and possible extension of the hematoma and progression of skin discoloration led to the decision for urgent surgical evacuation. Prognosis is generally favorable in stable patients, but severe cases can lead to major complications or death, with mortality rates up to 30% in high-risk groups ^(6)^. Clinicians should suspect RSH in patients with acute abdominal pain, abdominal wall discoloration, and severe coughing, even in the absence of anticoagulant therapy.

### Conclusions

This case illustrates that severe coughing can trigger RSH and cause hemodynamic instability. Clinicians should suspect RSH in patients with abdominal pain and abdominal wall discoloration after coughing, since early recognition and intervention are essential.

## Article Information

### Acknowledgments

We would like to thank the Japan Medical Communication for the English language editing.

### Author Contributions

Kazuhiko Iwasaki initiated the idea for case reporting and prepared a copy of the manuscript with Satoshi Watanabe and Seiji Yano. Kazuhiko Iwasaki was responsible for drafting and image modification. All of the authors have read and approved the final manuscript.

### Conflicts of Interest

None

### Ethics Approval and Consent to Participate

Informed consent for participation was obtained from the patient. The approval number from our Ethics Review Committee was 23-4.

### Consent for Publication

Written informed consent was obtained from the patient for publication of this case report and accompanying images. A copy of the written consent is available for review by the Editor-in-Chief of the journal.

### Availability of Data and Material

All data generated or analyzed during this study are included in this article. Further inquiries can be directed to the corresponding authors.
